# *Empty Pericarp24* and *Empty Pericarp25* Are Required for the Splicing of Mitochondrial Introns, Complex I Assembly, and Seed Development in Maize

**DOI:** 10.3389/fpls.2020.608550

**Published:** 2020-12-23

**Authors:** Zhihui Xiu, Ling Peng, Yong Wang, Huanhuan Yang, Feng Sun, Xiaomin Wang, Shi-Kai Cao, Ruicheng Jiang, Le Wang, Bao-Yin Chen, Bao-Cai Tan

**Affiliations:** ^1^Key Laboratory of Plant Development and Environmental Adaptation Biology, Ministry of Education, School of Life Sciences, Shandong University, Qingdao, China; ^2^Key Laboratory of Cell Activities and Stress Adaptations, Ministry of Education, School of Life Sciences, Lanzhou University, Lanzhou, China

**Keywords:** EMP24, EMP25, pentatricopeptide repeat protein, mitochondrion, seed development, maize

## Abstract

RNA splicing is an essential post-transcriptional regulation in plant mitochondria and chloroplasts. As the mechanism of RNA splicing remains obscure, identification and functional elucidation of new splicing factors are necessary. Through a characterization of two maize mutants, we cloned *Empty pericarp 24* (*Emp24*) and *Empty pericarp 25* (*Emp25*). Both *Emp24* and *Emp25* encode mitochondrion-targeted P-type PPR proteins. EMP24 is required for the splicing of *nad4* introns 1 and 3, which was reported (Ren Z. et al., 2019), and EMP25 functions in the splicing of *nad5* introns 1, 2, and 3. Absence of either Nad4 or Nad5 proteins blocks the assembly of mitochondrial complex I, resulting in the formation of a sub-sized complex I of similar size in both mutants. Mass spectrometry identification revealed that the subcomplexes in both mutants lack an identical set of proteins of complex I. These results indicate that EMP24 and EMP25 function in the splicing of *nad4* and *nad5* introns, respectively, and are essential to maize kernel development. The identification of the subcomplexes provides genetic and molecular insights into the modular complex I assembly pathway in maize.

## Introduction

Mitochondria are the main energy powerhouse of the cell, which perform oxidative phosphorylation through the electron transport chain (ETC) (Knoop, 2013). The cytochrome pathway of ETC is composed of four protein complexes, I, II, III, and IV. Complex I (NADH dehydrogenase) is the inception point of ETC, which functions to remove two electrons from NADH and transfers to ubiquinone (UQ). Complex II (succinate dehydrogenase) is a parallel electron transport pathway to complex I and delivers additional electrons to the quinone pool (Q). Complex III (cytochrome *c* reductase) transfers electrons from ubiquinol to cytochrome *c*, which is a water-soluble electron carrier. Complex IV (cytochrome *c* oxidase) removes electrons from cytochrome *c* to oxygen (O_2_) for water production (Sweetlove et al., 2007; Sazanov, 2015). In the ETC reactions, the protons are transported from the matrix to the intermembrane space, while complex V (ATP-synthase) pumps protons back to the matrix for adenosine triphosphate (ATP) synthesis, which keeps the balance of electron charge between the matrix and the intermembrane space (Zhou et al., 2015). The subunits of these complexes are encoded by both the mitochondrial and nuclear genome; thus, coordination between the two genetic systems is important (Poyton and McEwen, 1996).

As the descendant of α-proteobacteria endosymbiont, mitochondrion in higher plants retains its own genome, which encodes approximately 40 identified proteins (variable in different species) and numerous open-reading frames (ORFs) with unknown origin and function (Unseld et al., 1997; Adams et al., 2002; Notsu et al., 2002; Clifton et al., 2004). The expression of mitochondrial genes is regulated at the post-transcriptional level by nucleus-encoded factors through RNA splicing, cleavage, stabilization, and editing (Binder and Brennicke, 2003; Liere et al., 2011; Hammani and Giege, 2014). These processes are critical for mitochondrial functions as perturbations of these events usually result in defects in growth and development (Meyer et al., 2009; Liu et al., 2013), hormone response (Jiang et al., 2014; Sechet et al., 2015), and cytoplasmic male sterility (Hu et al., 2012; Tang et al., 2014).

Among the post-transcriptional regulation processes, RNA splicing of group II introns in higher plant mitochondria relies on the splicing factors due to the loss of self-splicing capacity of these introns (Bonen, 2008). Different from the splicing facilitated by specific maturase (Mat) that is encoded within the intron itself in bacterial and yeast mitochondria (Bonen and Vogel, 2001), the plant mitochondrial genes contain only one *MatR* in the *nad1* intron 4 and four MATs (nMAT) are encoded by the nucleus in Arabidopsis (Keren et al., 2009, 2012; Cohen et al., 2014). These maturases are required for the splicing of a large number of introns (Keren et al., 2009, 2012; Cohen et al., 2014; Sultan et al., 2016). Additionally, nucleus-encoded factors of different RNA-binding protein families have been identified to function in mitochondrial intron splicing. These include pentatricopeptide repeat (PPR) proteins, plant organellar RNA recognition (PORR) domain protein, mitochondrial transcription termination factor (mTERF), mitochondrial CAF-like splicing factor (mCSF), RNA DEAD-box helicases, and regulator of chromosome condensation (RCC1) protein (Brown et al., 2014).

The PPR protein family is remarkably large, >400 members in land plants. Its members are composed of up to 30 degenerate repeats of a 35-amino-acid motif arrayed in tandem (Small and Peeters, 2000) and are categorized into two major subfamilies according to the motif type. The P-subfamily consists of a canonical 35-residue motif (P-type), while the PLS-subfamily is composed of short (S), long (L), and P-type motifs often with additional C-terminal domains (E, E +, and DYW) (Lurin et al., 2004). Most PPR proteins are targeted to organelles to function in post-transcriptional regulation (Small and Peeters, 2000). However, owing to its large number, functions of many PPR proteins remain to be identified. In this study, we cloned *Empty pericarp 24* (*Emp24*) and *Empty pericarp 25* (*Emp25*) in mutants of maize seed development. EMP24 is the same as EMP602, which is required for the splicing of *nad4* introns 1 and 3 (Ren Z. et al., 2019), and EMP25 is required for the splicing of *nad5* introns. Lack of either Nad4 or Nad5 blocks the assembly of complex I holoenzyme but allows the formation of a sub-sized complex I containing N and Q modules in the matrix arm and proximal (P_P_) module modules in the membrane arm, which is similar to the complex I assembly in Arabidopsis (Ligas et al., 2019). Analysis of the subcomplex reveals that the absence of either Nad4 or Nad5 leads to the formation of a subcomplex that misses an identical set of subunits. Based on the proposed modular pathway of mitochondrial complex I assembly in Arabidopsis (Ligas et al., 2019), our results provide genetic evidence to the modular complex I assembly pathway in monocots.

## Materials and Methods

### Plant Materials

The *emp24* and *emp25* alleles were obtained from the UniformMu population, which was generated by introgressing *Mu* active line into the W22 inbred genetic background (McCarty et al., 2005). All plants were cultivated in the field under natural conditions.

### Light Microscopy of Cytological Sections

WT and mutant sibling kernels were harvested from *emp25* self-pollinated heterozygous ears at different days after pollination (DAP), respectively. The sections’ preparation, staining, and observation were performed as previously described (Shen et al., 2013).

### Gene Cloning and Linkage Analysis

The *Mu* inserted flanking sequences were isolated by the Mu-seq high-throughput sequencing approach (Liu et al., 2016). *emp24* and *emp25* were 2 of 14 independent mutants in the 14 × 12 grid. Linkage analysis was performed as previously described (Sun et al., 2015), using EMP24-R1 and TIR8 as primers (62°C annealing temperature running 8 cycles followed by 56°C annealing temperature running 32 cycles). The same PCR condition was used in *emp25* linkage analysis with primers EMP25-R1 and TIR8. The primers are listed in Supplementary Table S1.

### Subcellular Localization of EMP24 and EMP25

The full length of *Emp24* or the 669-bp (start from ATG) fragment of *Emp25* was ligated into pENTR/D-TOPO (Invitrogen, United States) and then introduced into binary vector pBI221 (CaMV *35S* promoter) and pGWB5 (CaMV *35S* promoter) via Gateway technology, respectively. The mitochondria-localized protein ATPase was fused with RFP in the C-terminal in the 326-RPF vector (CaMV *35S* promoter). EMP24-GFP and ATPase-RFP were co-transiently expressed in *Arabidopsis thaliana* leaf protoplast by *Agrobacterium tumefaciens* (EHA105) (Yoo et al., 2007). EMP25-GFP was transiently expressed in *Nicotiana tabacum* leaves by *A. tumefaciens* (EHA105) infiltration (van Herpen et al., 2010). The fluorescence signals were detected by a ZEISS LSM 700 confocal microscope. Mitochondria were labeled by the MitoTracker Red (Life Technologies, United States).

### RNA Extraction, RT-PCR, Quantitative RT-PCR, and cRT-PCR

Total RNA was extracted with the Trizol reagent (Invitrogen, United States) and treated with RNase-free DNase I (NEB, United States) to eliminate g-DNA contamination and then reverse transcribed using random hexamers as primers. RT-PCR analysis of full-length transcript level difference in *emp25* with sibling WT in 34 protein-coded maize mitochondrial genes was performed with *ZmActin* (ZmActin-F1/R1) as normalization. The mitochondrial gene primers were used as previously described (Liu et al., 2013). In the RT-PCR of *nad5* splicing analysis, four pairs of primers (nad5-F1/R1, nad5-F2/R2, nad5-F3/R3, and nad5-F4/R4) were designed to test four introns splicing, respectively. PCR conditions were 95°C for 30 s, 56°C for 30 s, and 72°C for 120 s, with a total of 30 cycles. *ZmActin* (Zmactin-F1/R1) was used as normalization. Primers for qRT-PCR analysis were designed according to Colas des Francs-Small et al. (2014). qRT-PCR was performed as described above.

Primers for quantitative RT-PCR (qRT-PCR) analysis were designed according to Colas des Francs-Small et al. (2014). cDNAs were obtained from three biological repeats, respectively. qRT-PCR was performed using SYBR Green on a LightCycler^®^ 96 (Roche). PCR conditions are 95°C for 10 s and 60°C for 10 s, with a total of 40 cycles, following 65°C for 5 s and 95°C for 5 s for melt curve. qRT-PCR was performed as described above. Circular RT-PCR (cRT-PCR) analysis was performed as previously described (Zhang et al., 2019). All primers are listed in Supplementary Table S1.

### Editing Analysis of *nad4* and *nad5* mRNA in WT and *emp24*/*emp25*

Total RNA was extracted with the Trizol reagent (Invitrogen, United States) and treated with RNase-free DNase I (NEB, United States) to eliminate g-DNA contamination and then reverse transcribed using random hexamers as primers. The full length of *nad4* and *nad5* was amplified by using primers *nad4*-F1/R1 and *nad5*-F1/R1, respectively. An RNA editing analysis was conducted from these samples by directly sequencing *nad4* and *nad5*, as described in Liu et al. (2013).

### Mitochondrial Complex I Activity and Western Blotting Assay

Crude and intact mitochondria were isolated from about 10 ml of maize kernel (removed pericarp) at 13 DAP. Blue native PAGE and in-gel complex I activity assay were performed as previously described (Meyer et al., 2009). Protein concentration was determined using the Bradford assay (Bio-Rad). Western blotting was performed as previously described (Sun et al., 2015).

## Results

### Molecular Cloning of *Emp24* and *Emp25*

The high-throughput *Mu*-seq analysis was employed to isolate the causal gene of *emp24* and *emp25* (McCarty et al., 2013). After all germinal insertions are distributed into samples in the grid (14 × 12) based on barcodes, the phenotype of *emp24-1* was found to co-segregate with the *Mu* insertion at +45 bp start from ATG of *GRMZM2G464510* (Supplementary Figure S1A). Linkage analysis by using *GRMZM2G464510* gene-specific primer with Mu primer TIR8 showed a tight linkage in 224 individual plants in an F2 progeny of *emp24-1* mutant (Supplementary Figure S1B). To confirm that the *emp24-1* phenotype is due to mutation of *GRMZM2G464510*, we isolated two alleles with *Mu* inserted in the coding region of *GRMZM2G464510* from Maize Genetics Cooperation Stock Center, UFMu08492 and UFMu01097. The *Mu* transposon insertion position of UFMu08492 (+ 45 bp start from ATG) is identical to the one in *emp24-1*, and the UFMu01097 with *Mu* inserted at + 483 bp (start from ATG) was named *emp24-2*. Self-pollinated heterozygotes for *emp24-2* segregated *emp* kernels in a recessive manner, and crosses between *emp24-1* and *emp24-2* produced *emp* kernels (Supplementary Figure S2A). These results confirmed that *GRMZM2G464510*, which has been identified as *Emp602* encodes a P-type PPR protein that is specific for the splicing of mitochondrial *nad4* introns 1 and 3 (Ren Z. et al., 2019), is the causal gene for the *emp24* phenotype.

For *emp25-1*, the *Mu*-Seq analysis identified a *Mu* insertion at +98 bp start from ATG of *GRMZM2G312954* (Supplementary Figure S1C). Linkage analysis in 147 members of *emp25-1*/ + selfed progeny by using TIR8 and *GRMZM2G312954* gene specific primers found a tight linkage between the *Mu* insertion and the *emp* phenotype (Supplementary Figure S1D). A second allele was identified in UFMu-02672 (+88 bp start from ATG) from the Maize Stock Center, referred to as *emp25-2*. Crossed ears between *emp25-1*/+ and *emp25-2/*+ exhibited *emp* kernels (Supplementary Figure S2B). These results show that *GRMZM2G312954* is the causal gene for the *emp25* phenotypes, referred to as *Emp25* hereafter.

### Loss of Function in *Emp25* Impairs Embryo and Endosperm Development

**FIGURE 1:**
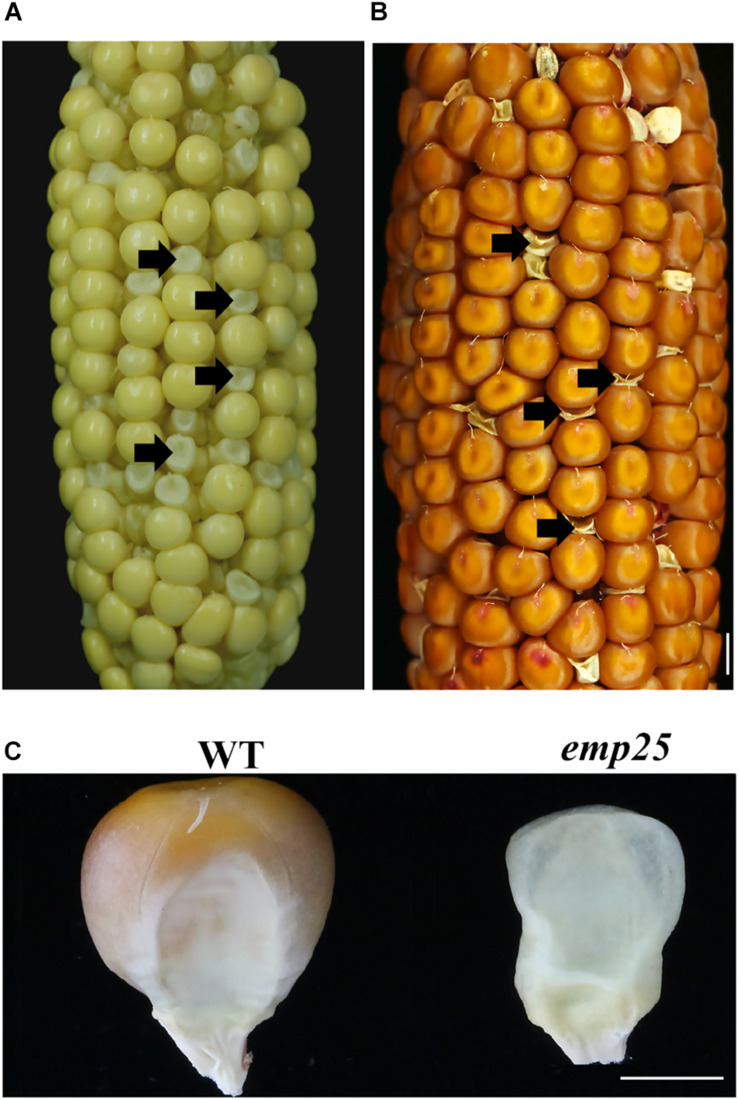
Phenotype of *emp25.* **(A,B)** The ear segregates the *emp25* kernels at 11 days after polination (DAP) **(A)** and maturity **(B)**. Arrows indicate the mutants. Scale bar = 0.5 cm. **(C)** Germinal side view of the WT and *emp25* kernel. Scale bar = 0.2 cm.

The *emp25* mutant showed a 3:1 segregation ratio of wild type (WT) and empty pericarp kernels in self-pollinated heterozygous plants, indicating that the mutation is monogenetic and recessive. The mutant kernels of *emp25* could be clearly identified at 11 DAP (Figure 1A), and at maturity, the pericarp collapsed and wrinkled, giving rise to an *emp* phenotype (Figures 1B,C).

**FIGURE 2:**
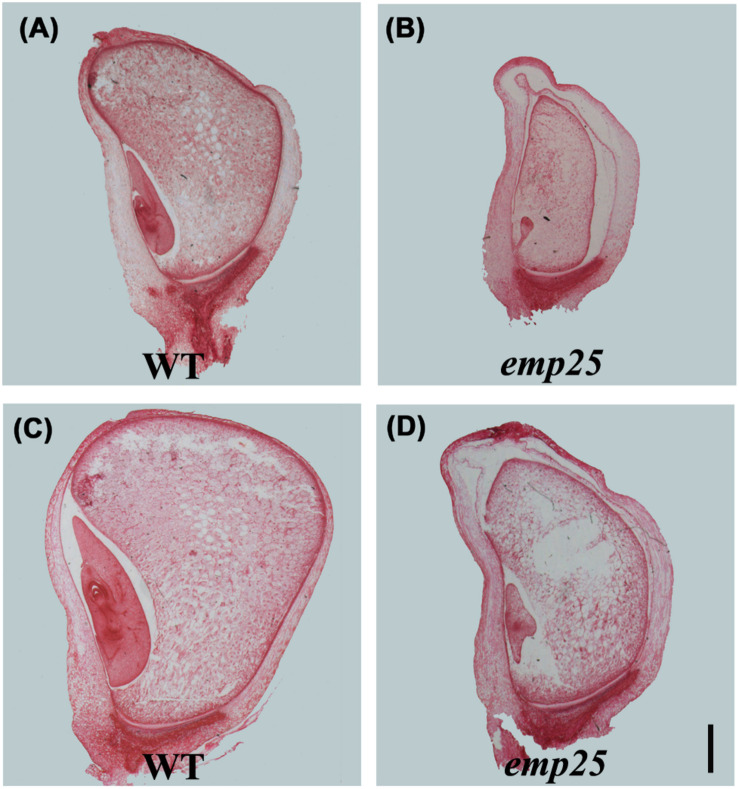
Embryo and endosperm development of *emp25.* Paraffin sections of the *emp25* mutant kernels and the corresponding WT siblings at 10 DAP **(A,B)** and 14 DAP **(C,D)**. Scale bar = 1 mm.

The embryo development of maize is divided into transition, coleoptilar, and late embryogenesis stage (Fontanet and Vicient, 2008). The endosperm development in maize is classified into coenocytic, cellularization, differentiation, and maturation stages (Olsen, 2001). To underpin the developmental arrest, the *emp25* and the WT sibling kernels in the same ear were analyzed at different DAP by paraffin section. At 10 DAP, the WT kernels differentiated clear coleoptile, shoot apical meristem, and suspensor, but the *emp25* mutant just formed an embryo proper attached to the suspensor (Figures 2A,B). At 14 DAP, the WT embryo reached late embryogenesis stage with primary leaves, but the *emp25* mutant embryo reached the transition stage (Figures 2C,D) and remained at that stage after, suggesting that the embryo development of *emp24* was arrested at the transition stage. Both the WT and the *emp25* mutant endosperm finished the cellularization and reached the differentiation stage at 14 DAP, but the smaller size of mutant endosperm compared with WT endosperm and the hollow between endosperm and pericarp in mutant suggested that endosperm development was seriously affected in the *emp25* (Figures 2B,D).

### EMP25 Is Localized in Mitochondria

**FIGURE 3:**
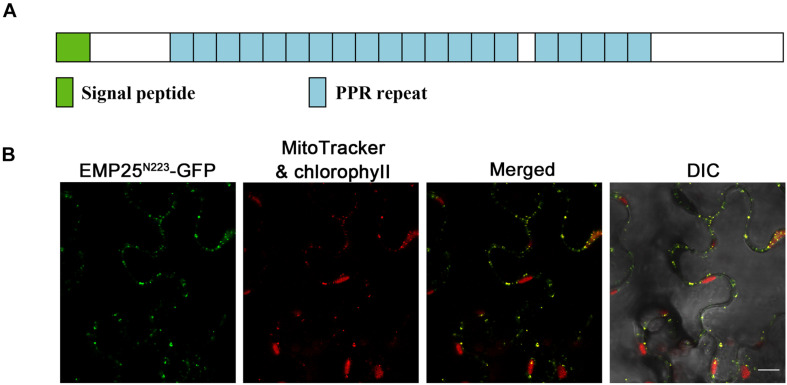
EMP25 is a mitochondrion-localized P-type PPR protein. **(A)** Schematic diagram of the EMP25 protein. **(B)** Subcellular localization of EMP25. The N-terminus (223 amino acids) of EMP25 was fused with the green fluorescence protein (GFP). The EMP25^N223aa^–GFP fusion was transiently expressed in tobacco leaf epidermal cells and the signal was detected by confocal fluorescent microscopy. Mitochondria were detected with MitoTracker. DIC, differential interference contrast. Scale bar = 10 μm.

The *Emp25* gene contains five introns (Supplementary Figure S1C), also encoding a P-type PPR protein with 20 PPR motifs (Figure 3A). EMP25 shares a 92% identity with Sb02g380100 in *Sorghum bicolor*, 70% identity with Os05g19390 in *Oryza sativa*, and 41% identify with At1g19290 (TANG2, Colas des Francs-Small et al., 2014) in *A. thaliana*. We tested the expression of *Emp25* in the mutant alleles by RT-PCR. No WT transcript was detected in two alleles (Supplementary Figure S3A). Also, the transcript level of *Emp25* in main tissues during maize growth and development was analyzed by qRT-PCR. Expression was relatively higher in shoot, pollen, leaf, and roots than in bract and tassel. During kernel development, *Emp25* is expressed at a higher level in the early developing kernels at 9 DAP than at later stages (Supplementary Figure S3B). These data indicate that *Emp25* is a ubiquitously expressed gene, rather than a seed-specific gene.

Because full EMP25-GFP fusion did not produce detectable signals, we fused the N-terminal 223 amino acids of EMP25 with GFP (GFP at the C-terminal). The fusion protein was transiently expressed in tobacco leaf epidermal cells and the MitoTracker red was used as the mitochondrial marker. The results showed that the GFP signals in small dots were completely merged with the MitoTracker red signals (Figure 3B), indicating that EMP25 is localized in mitochondria.

### The *emp25* Mutant Is Deficient in the Splicing of *nad5* Introns

**FIGURE 4:**
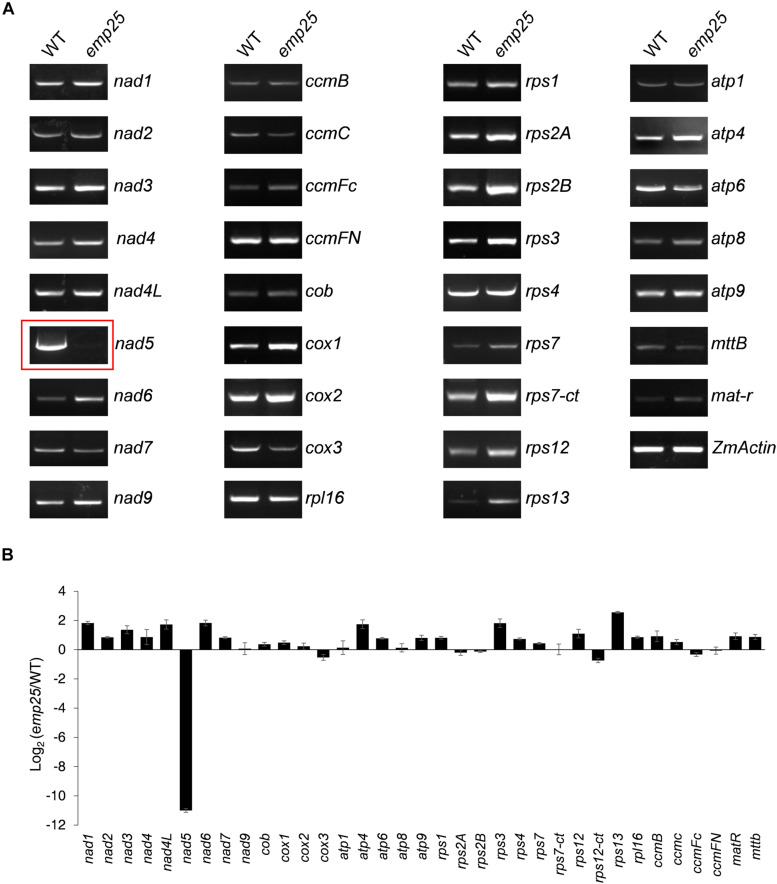
Analysis of mitochondrial gene transcripts in the *emp25* kernels. **(A)** RT-PCR analysis of the 34 mitochondrial protein coding genes in *emp25* with corresponding WT siblings. **(B)** qRT-PCR analysis of the mitochondrial transcripts in WT and *emp25.* Values represent the mean and standard deviation of three biological replicates. RNA was extracted from 13 DAP kernels without pericarp and was normalized against *ZmActin*.

Mitochondrial localized P-type PPR proteins have been shown to function in intron splicing (Barkan and Small, 2014). To uncover the functions of EMP25, we first examined the transcript levels of the 34 mitochondrial protein-coding genes in kernels at 13 DAP (pericarp removed) (Clifton et al., 2004). The primers were designed to amplify nearly full-length transcripts and the RNA templates were treated with DNase and normalized against *ZmActin* (GRMZM2G126010) (Li et al., 2014). Among the 34 genes, no significant difference in transcript levels was detected in most genes between WT and mutants. However, for *emp25*, the *nad5* transcript is missing (Figure 4A). Such results were consistent with the mRNA accumulation analysis of these 34 genes by qRT-PCR (Figure 4B).

**FIGURE 5:**
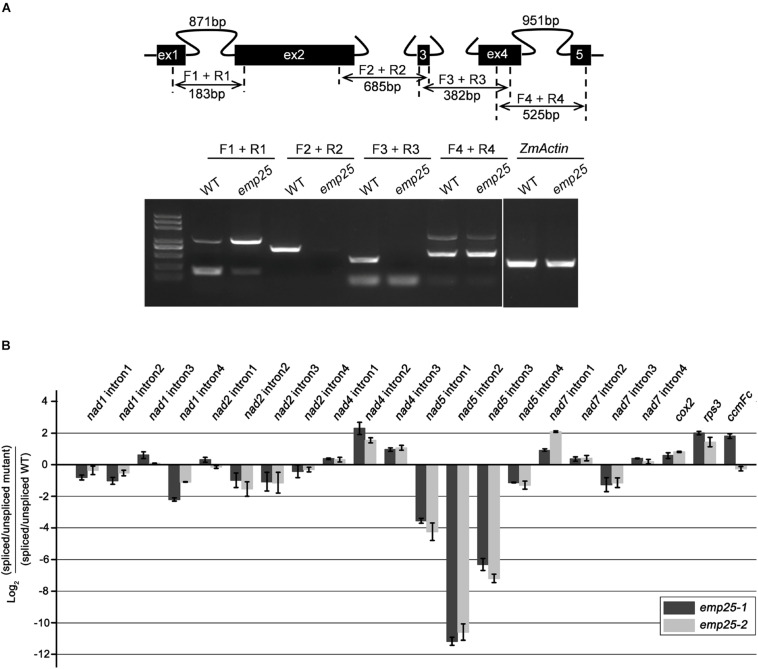
EMP25 is required for the splicing of *nad5* introns. **(A)** Schematic diagram of the maize mitochondrial *nad5* gene and RT-PCR analysis of intron splicing efficiency in the WT and *emp25*. Introns 1 and 4 are *cis*-splicing introns. Introns 2 and 3 are *trans*-splicing introns. The expected amplification products using different primer pairs are indicated. The arrows indicate the unspliced fragments of introns 1 and 4 (*cis*-splicing). **(B)** Quantitative RT-PCR (qRT-PCR) analysis of the 22 group II intorns in maize mitochondrial genes in two alleles of *emp25*. Values represent the mean and standard deviation of three biological replicates.

The maize mitochondrial *nad5* contains four introns, two *cis*- and two *trans*-introns (Figure 5A). To test whether the absence of mature transcripts is due to defects in intron splicing, we analyzed the splicing efficiency of each intron by RT-PCR with specific primers anchored across each intron. In *emp25*, the splicing of *nad5* intron 1 (*cis*) was drastically decreased, and the splicing of *nad5 trans*-introns 2 and 3 was abolished. For unspliced *cis*-introns, accumulation of a larger size fragment was detected, which is the unspliced intron as confirmed by sequencing. For *trans*-intron, such a large fragment was not detected, suggesting that no spliced transcript is produced in the mutants. To independently verify the splicing defects in the *emp25*, we determined the splicing efficiency of the mitochondrial 22 introns in maize by qRT-PCR analysis in two alleles. The result showed that the splicing efficiency of *nad5* introns 1, 2, and 3 was decreased 16-, 2, 000-, and 128-fold in the *emp25* mutant when compared to the WT (Figure 5B). Furthermore, in order to confirm that EMP25 is specific in intron splicing of *nad5*, we detected the editing sites and 5′/3′ maturation of *nad5* in *emp25*. No editing sites were changed (Supplementary Figure S4), and 5′/3′ maturation of *nad5* is normal in *emp25* (Supplementary Figure S5). These results confirmed that the loss of function in EMP25 affects the splicing of *nad5* introns 1, 2, and 3 in mitochondria.

**FIGURE 6:**
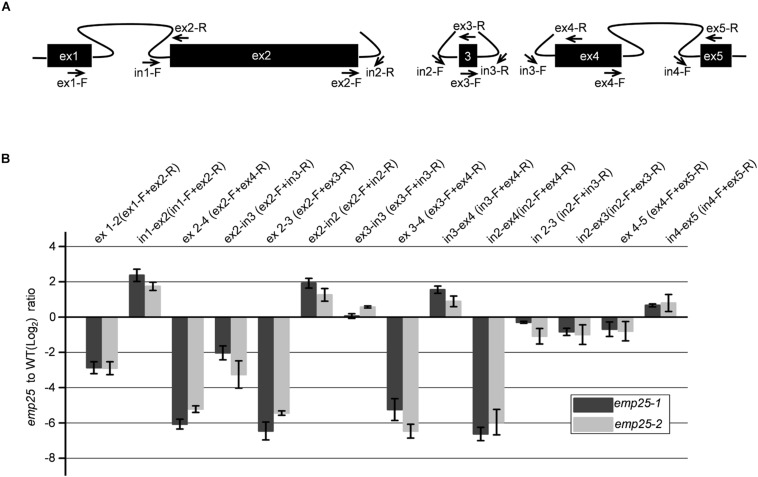
The splicing of *nad5* introns 1, 2, and 3 is impaired in *emp25.* **(A)** Diagram of the splicing events of *nad5* transcript and positions of the primers used. **(B)** qRT-PCR analysis of the mature and unspliced transcripts of individual *nad5* exons in *emp25* two alleles. Values represent the mean and standard deviation of three biological replicates.

The *nad5* gene in maize contains a 22-bp exon 3 (22 bp) adjacent to two *trans*-splicing introns 2 and 3 (Clifton et al., 2004; Figure 6A). It was reported that the *trans*-splicing of *nad5* intron 3 must occur before the *trans*-splicing of intron 2 or it will generate a variety of mis-spliced products of intron 2, which also results in the accumulation of unspliced fragments of intron 2 (Elina and Brown, 2010; Brown et al., 2014; Colas des Francs-Small et al., 2014). To determine whether the accumulation of unspliced intron 2 is due to the dysfunctional EMP25 or mis-splicing, we quantitated the splicing intermediates of the *nad5* transcripts with primers as previously described (Figure 6A; Colas des Francs-Small et al., 2014). The accumulation levels of both ex 2–3 and ex 2–4 decreased in *emp25*, and the result of ex2-in3, which indicates the accumulation level of mis-spliced fragments, also decreased in *emp25* (Figure 6B). These results, coupled with low splicing efficiency of introns 2 and 3 of *nad5* (Figure 5B), confirm the true splicing defects of *nad5* in *emp25*.

### Loss of Function in EMP24 and EMP25 Affects Mitochondrial Complex I Assembly and Activity

**FIGURE 7:**
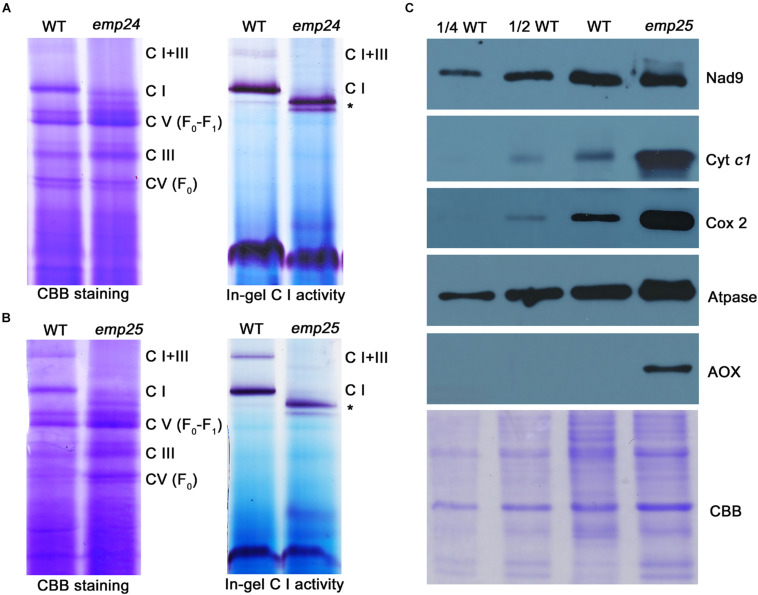
Defect in the complex assembly and activity in *emp24* and *emp25.* **(A,B)** The blue native polyacrylamide gel electropheresis (BN-PAGE) gel was stained with Coomasssie Brilliant Blue (CBB). In-gel NADH dehydrogenase activity assay of complex I used dihydrolipoamide dehydrogenase (DLDH) as a loading control. C I: Complex I; C III: Complex III; C V: Complex V; *: sub-complex I detected in mutant. **(C)** Crude mitochondrial extracts from endosperm and embryo of *emp25* with sibling WT kernels were used for Western blot analysis with antibodies against Nad9, Cyt *c1*, Atpase, Cox2, and AOX. CBB staining was used as a loading control.

Nad4 and Nad5 are the subunits of complex I in the mitochondrial electron transfer chain (Colas des Francs-Small et al., 2014; Subrahmanian et al., 2016). The complete deficiency in *nad4* intron splicing in *emp24* and *nad5* intron splicing in *emp25* causes absence of Nad4 and Nad5 proteins. These mutants provide a good genetic model to test the role of each subunit in the assembly and function of the complex. By using blue native polyacrylamide gel electrophoresis (BN-PAGE) and in-gel assay of the NADH dehydrogenase activity, we analyzed the abundance and activity of the complexes in *emp24* and *emp25*. In the Coomassie blue staining, *emp24* and *emp25* showed a similar profile of the complexes. Compared with the WT, complex I (CI) and supercomplex I + III (CI + III) were absent in both mutants, and complex III (CIII) and V (CV) were slightly increased (Figures 7A,B). Instead, two subcomplexes smaller than CI appeared in the mutants. Subsequent in-gel NADH dehydrogenase activity staining showed that the two smaller complexes have the activity, indicating that they are CI subcomplexes, hence designated as CI′ and CI″ (Figures 7A,B). Such results of *emp24* were consistent with the reports in *emp602* (Ren Z. et al., 2019). We also surveyed the protein abundance of the representative subunit of the mitochondrial complexes by Western blot analysis in *emp25*. Compared to the WT, Nad9 (CI) was slightly increased, whereas Cytc1 (CIII) and ATPase-A (CV) were dramatically increased in *emp25* (Figure 7C). This result is consistent with the BN-PAGE analysis. In addition, the assembly of CIII, CIV, and CV was complete using representative antibody blot after transferring membrane from BN-gel (Supplementary Figure S6A) and respiratory rate was dramatically decreased in *emp25* (Supplementary Figure S6B). These results indicated that absence of Nad4 or Nad5 protein as a result of loss-of-function mutation in EMP24 or EMP25 impairs the assembly and activity of mitochondrial complex I.

The formation of subcomplexes in the *emp24* and *emp25* mutants provided an opportunity to investigate the role of Nad4 and Nad5 in complex I assembly. We collected the CI′ and CI″ band from the BN-PAGE in two mutants, respectively, and subjected it to mass spectrometry analysis. The CI in the WT was analyzed as a reference. As shown in Table 1, 46 proteins were identified in the CI complex in the WT, but the *emp24* and *emp25* mutants showed identical deficiency in 11 proteins, i.e., Nad4, Nad5, NADH-ubiquinone dehydrogenase 1 beta subcomplex subunit 2, 3-A, 4, 7, 8, 9, 10, and 11 (also named NDUFB2, 3, 4, 7, 8, 9, 10, and 11), and NADH-ubiquinone dehydrogenase 1 subunit C1 (NDUFC1/KFYI) (Liu et al., 2019). These results showed that the lack of either Nad4 or Nad5 blocks the assembly of complex I and its activity, resulting in the formation of subcomplexes. Surprisingly, absence of Nad4 or Nad5 leads to the formation of a subcomplex lacking an identical set of proteins.

**TABLE 1:** Protein mass spectrometry analysis of complex I subunits in *emp24* and *emp25.*

Accession number	Subunit	Protein description	WT		*emp24*		WT		*emp25*	
			P	S	P	S	P	S	P	S
ZeamMp017	Nad1	NADH dehydrogenase subunit 1	3	9.8	2	4.5	4	23.1	4	16.8
amMp016	Nad2	NADH dehydrogenase subunit 2	4	22.4	5	26.7	7	32.9	4	17.2
ZeamMp093	Nad3	NADH dehydrogenase subunit 3	1	3.8	1	4.5	1	4.1	1	4.2
ZeamMp012	Nad4L	NADH dehydrogenase subunit 4L	N	N	N	N	N	N	N	N
ZeamMp032	Nad4	NADH dehydrogenase subunit 4	2	8.6	N	N	2	9.4	N	N
ZeamMp071	Nad5	NADH dehydrogenase subunit 5	5	16.3	N	N	8	30.1	N	N
ZeamMp185	Nad6	NADH dehydrogenase subunit 6	2	7.4	3	10.3	4	12.2	3	8.6
ZeamMp086	Nad7	NADH dehydrogenase subunit 7	8	43.8	7	36.9	12	62.5	7	37.4
ZeamMp036	Nad9	NADH dehydrogenase subunit 9	15	123.1	16	114.1	17	171.4	13	96.4
GRMZM2G163468	NDUFA1/MFWE	NADH-ubiquinone dehydrogenase 1 a-subcomplex subunit 1	2	5.3	1	5.0	4	34.5	2	4.5
GRMZM2G079746	NDUFA3/B9	NADH-ubiquinone dehydrogenase 1 a-subcomplex subunit 3	1	3.1	1	2.1	1	6.6	1	4.0
ZM2G014382	NDUFA6/B14	NADH-ubiquinone dehydrogenase 1 a-subcomplex subunit 6	5	97.6	7	106.1	12	217.7	8	92.5
GRMZM2G074028	NDUFA7/B14.5a	NADH-ubiquinone dehydrogenase 1 a-subcomplex subunit 7	10	66.4	8	48.4	11	92.5	9	49.2
GRMZM2G006085	NDUFA8/PGIV	NADH-ubiquinone dehydrogenase 1 a-subcomplex subunit 8-B	4	14.8	4	13.4	4	26.4	4	25.5
GRMZM2G143651	NDUFA9/B22	NADH-ubiquinone dehydrogenase 1 a-subcomplex subunit 9	20	173.66	19	140.2	24	256.6	18	137.8
GRMZM2G038375	NDUFA11/B17.4	NADH-ubiquinone dehydrogenase 1 a-subcomplex subunit 11	6	39.8	1	4.0	5	44.0	1	4.3
GRMZM2G330213	NDUFA12/B17.2	NADH-ubiquinone dehydrogenase 1 a-subcomplex subunit 12	12	72.0	16	79.0	17	129.1	9	61.5
GRMZM2G070716	NDUFA13/A13	NADH-ubiquinone dehydrogenase 1 a-subcomplex subunit 13-A	8	64.5	8	60.4	13	124.8	6	55.9
GRMZM2G117811	NDUFB1/MNLL	NADH-ubiquinone dehydrogenase 1 b-subcomplex subunit 1	3	27.2	2	17.6	3	30.6	3	29.8
GRMZM2G106607	NDUFB2/AGGG	NADH-ubiquinone dehydrogenase 1 b-subcomplex subunit 2	4	12.7	N	N	5	30.4	N	N
GRMZM2G421234	NDUFB3/B12	NADH-ubiquinone dehydrogenase 1 b-subcomplex subunit 3-A	3	25.2	N	N	3	20.0	N	N
GRMZM2G004111	NDUFB4/B15	NADH-ubiquinone dehydrogenase 1 b-subcomplex subunit 4	3	19.0	N	N	6	30.0	N	N
GRMZM2G137312	NDUFB7/B18	NADH-ubiquinone dehydrogenase 1 b-subcomplex subunit 7	6	27.3	N	N	7	44.9	N	N
AC234161.1_FGP010	NDUFB8/ASHI	NADH-ubiquinone dehydrogenase 1 b-subcomplex subunit 8	5	10.4	N	N	5	20.5	N	N
GRMZM2G115621	NDUFB9/B22	NADH-ubiquinone dehydrogenase 1 b-subcomplex subunit 9	8	54.3	N	N	11	82.8	N	N
GRMZM2G456603	NDUFB10/PDSW	NADH-ubiquinone dehydrogenase 1 b-subcomplex subunit 10	9	63.6	N	N	15	130.0	N	N
GRMZM2G132748	NDUFB11/ESSS	NADH-ubiquinone dehydrogenase 1 b-subcomplex subunit 11	3	28.0	N	N	3	52.9	N	N
GRMZM2G056606	NDUFC1/KFYI	NADH-ubiquinone dehydrogenase 1 subunit C1	1	11.3	N	N	1	12.6	N	N
GRMZM2G313672	NDUFC2/B14.5b	NADH-ubiquinone dehydrogenase 1 subunit C2	2	11.6	2	5.8	2	18.5	2	11.9
GRMZM2G112079	NDUFB5/SGDH	NADH-ubiquinone dehydrogenase 1 b-subcomplex subunit 5	6	59.2	3	29.7	7	158.3	2	31.9
GRMZM2G040209	NDUFV1/51kD	NADH-ubiquinone dehydrogenase flavoprotein 1	32	249.25	32	225.9	41	389.6	30	266.1
GRMZM2G067992	NDUFV2/24kD	NADH-ubiquinone dehydrogenase flavoprotein 2	20	134.9	18	137.1	24	260.8	16	96.7
GRMZM2G145854	NDUFS1/75kD	NADH-ubiquinone dehydrogenase iron-sulfur protein 1	33	337.6	36	308.4	42	455.2	33	296.0
ZM2G105207	NDUFS4/AQDQ	NADH-ubiquinone dehydrogenase iron-sulfur protein 4	9	28.9	6	24.9	12	50.1	7	24.5
M2G149414	NDUFS5/15kD	NADH-ubiquinone dehydrogenase iron-sulfur protein 5	5	29.7	7	39.4	10	60.15	7	50.2
GRMZM2G171236	NDUFS6/13A	NADH-ubiquinone dehydrogenase iron-sulfur protein 6	3	15.8	3	12.9	8	24.1	4	11.3
GRMZM2G042034	NDUFS7/PSST	NADH-ubiquinone dehydrogenase iron-sulfur protein 7	11	95.4	8	75.4	11	131.5	9	80.5
G089713	NDUFS8/TYKY	NADH-ubiquinone dehydrogenase iron-sulfur protein 8-B	12	50.3	15	67.8	16	104.4	13	66.4
GRMZM2G008464	NDUFA2/B8	NADH-ubiquinone oxidoreductase 10.5 kDa subunit	9	62.1	8	57.2	10	96.9	10	56.4
2G125668	NDUFA5/AB13	NADH ubiquinone oxidoreductase 29 kDa subunit	14	106.2	14	75.1	14	157.9	12	70.4
GRMZM2G140885	CA1	Carbonic anhydrase 1	12	104.5	12	112.1	18	213.8	11	127.1
GRMZM2G037177	CA2	Carbonic anhydrase 2	15	129.2	12	136.2	213.8	18	11	145.2
GRMZM2G125668	CAL1	Carbonic anhydrase-like 1	8	87.5	7	72.3	267.6	21	13	66.6
GRMZM2G123966	CAL2	Carbonic anhydrase-like 2	6	64.5	5	62.5	118.1	9	6	39.1
GRMZM2G469969	GLDH1	L-galactono-1 4-lactone dehydrogenase	6	12.8	46	292.2	110.2	10	4	305.8
GRMZM5G898597	P1/11kD	/	9	67.1	8	59.2	21.5	9	40	52.1
GRMZM5G801031	P2/16kD	/	6	48.4	5	52.9	103.4	9	8	50.2

*P, Peptides; S, Score; N, None.*

## Discussion

### EMP24 and EMP25 Function in the Splicing of *nad4* and *nad5* Introns, Respectively, in Mitochondria

This study has cloned *Emp24* and *Emp25*. The correct cloning of the genes is confirmed by molecular and genetic analysis of independent alleles (Supplementary Figures S1, S2). *Emp24* has been identified as *Emp602* that encodes a mitochondrion-localized P-type PPR protein, which functions in the splicing of *nad4* introns 1 and 3 (Ren Z. et al., 2019). *Emp25* encodes a P-type PPR protein targeted to mitochondria that is required for the splicing of nad5 introns 1, 2, and 3 (Figure 3). EMP25 shares a 41% identity with the Arabidopsis TANG2; the function of these two proteins is either distinct or diverged. Loss of function of TANG2 affects the splicing of introns 1, 2, and 2–3, whereas TANG2 is required mainly for the splicing of *nad5* intron 3 in Arabidopsis, and the reduced splicing efficiency of *nad5* introns 1 and 2 could be indirectly influenced by intron 3 splicing defects (Colas des Francs-Small et al., 2014). Although mutation of *Emp25* also exhibited defects in the splicing of *nad5* introns 1, 2, and 2–3, the splicing of intron 2 shows more serious defects compared with intron 3 (Figures 5, 6). It is possible that TANG2 and EMP25 are derived from an ancestor protein that is originally recruited for the splicing of *nad5* intron 3. During evolution, however, mutations in the maize *nad5* introns 1 and 2 promoted the recruitment of EMP25 for efficient splicing, whereas in Arabidopsis, either different mutations occurred or other proteins were recruited for the splicing of *nad5* introns 1 and 2. Thus, the true ortholog of EMP25 cannot be identified in Arabidopsis, reflecting that both the introns and the PPR proteins co-evolve fast to maintain efficient splicing.

P-type PPR proteins have been reported in the splicing of mitochondrial and chloroplast introns. In Arabidopsis, OTP43 functions in the splicing of *nad1* intron 1 (de Longevialle et al., 2007), TANG2 functions in the splicing of *nad5* intron 3, and OTP439 functions in the splicing of *nad5* intron 2 (Colas des Francs-Small et al., 2014). These proteins are all P-type PPR proteins. In maize, P-type PPR proteins EMP10, EMP16, DEK2, and DEK35 have functions in the splicing of mitochondrial introns (Chen et al., 2016; Xiu et al., 2016; Cai et al., 2017; Qi et al., 2017). As some 20 introns exist in the plant mitochondrial genes, EMP24 and EMP25 contribute to the effort of identification of intron splicing factors in mitochondria. The mechanism by which plant organellar group II introns are spliced is not fully understood yet despite the fact that a number of RNA-binding proteins are found to participate in this process. Group II introns can self-splice, but introns in plant organelles have lost the self-splicing activity due to the mutations that accumulated during evolution. It is hypothesized that PPRs and other RNA-binding proteins are recruited to facilitate the formation of such a catalytic active conformation of the intron. Based on this idea, we predicted the EMP25 putative binding sites (as indicated in Supplementary Figure S7) based on the proposed 6, 1′ binding codes of the PPR motif (Barkan et al., 2012; Takenaka et al., 2013; Yagi et al., 2013). Further analysis indicates that these sites are uniquely present in the three introns that require EMP25, but absent in other introns or/and exons in the maize mitochondrial genes. We speculate that these sites may be the cognitive recognition sequences of EMP25. However, as we did not perform the binding assay because of the difficulty in expressing EMP25 recombinant protein, further analysis is necessary.

### EMP24 and EMP25 Are Essential for Maize Seed Development

The loss of *Emp24* and *Emp25* function results in a similar phenotype with arrested embryo and endosperm development in maize (Figure 2). The empty pericarp phenotype highlights the importance of the ETC to seed development. As the powerhouse of the cell, mitochondria provide energy and metabolites to all cellular processes. The mutation in *emp24* and *emp25* eliminates the synthesis of Nad4 and Nad5, respectively, and blocks the assembly of mitochondrial complex I. As the first and the largest complex of the ETC, complex I transfers electrons onto ubiquinone and pumps protons from the matrix to the inner membrane for proton gradient in ATP synthesis (Brandt, 2006; Hirst, 2013). Deficiency of complex I impairs the function of ETC, disturbs the citric acid (TCA) cycle, and subsequently blocks the energy and intermediate supply, causing arrest of both embryo and endosperm development in the mutant.

Dysfunction in the mitochindrial complexes arresting seed development has been reported in a number of *emp/dek* mutants (Gutierrez-Marcos et al., 2007; Wang et al., 2017; Yang et al., 2017; Li et al., 2018; Sun et al., 2019). For example, *dek43* are deficient in the splicing of *nad4* introns and block complex I assembly and activity (Ren R. C. et al., 2019). Mutation in *emp21* conditions editing defects in multiple sites of mitochondrial genes and hinders the biogenesis of complexes I and V (Wang et al., 2019). PPR78, which functions in the maturation and stability of mitochondrial *nad5*, is required for the complex I assembly and seed development (Zhang et al., 2017). The mutation of *emp24* and *emp25* also disturbs complex I function and subsequently impairs the seed development.

### Analysis of the *emp24* and *emp25* Mutants Provides Genetic Insights Into the Assembly Pathway of Complex I

As important subunits of the mitochondrial complex I, the lack of Nad4 or Nad5 allowed the formation of smaller subcomplex I (CI′) in both the *emp24* and *emp25* mutants (Figures 7A,B). Analysis of the proteins in CI′ reveals that the subcomplexes in *emp24* and *emp25* are identical in subunit composition and uniformly lack 11 proteins including Nad4; Nad5; NDUFB2, 3, 4, 7, 8, 9, 10, and 11; and NDUFC1 (Table 1). This result indicates that the absence of Nad4 or Nad5 affects complex I assembly in the same way.

Mitochondrial complex I is an L-shaped holoenzyme with a hydrophobic arm embedded in the cristae membrane and a hydrophilic arm protruding to the matrix. Nad5 is located at the end of the hydrophobic arm, adjacent to Nad4 (Zhu et al., 2016; Liu et al., 2019), and interestingly, all the subunits missing in the *emp24* and *emp25* mutants are located at the end of the arm, suggesting that the absence of either Nad4 or Nad5 inhibits the assembly of these proteins into complex I.

It is proposed that the assembly of mitochondrial complex I follows a modular pathway in which subunits initially form subcomplexes and then assemble into the holocomplex I (Subrahmanian et al., 2016; Ligas et al., 2019). These subcomplexes are defined as N module (NADH binding), Q module (electrons transfer), and P module (peripheral arm), which is further divided into P_P_ module (proximal section) and P_D_ module (distal section) (Klodmann et al., 2010). The analysis of the complex assembly in *emp24* and *emp25* provides genetic evidence that Nad4 and Nad5 are the core members of the P_D_ module because deficiency of either protein hinders the assembly. This analysis indicates that nuclear mutants affecting mitochondrial protein expression are valuable genetic materials for the study of mitochondrial complex assembly and other processes, circumventing the inability of creating mitochondrial gene mutants due to cell lethality.

## Data Availability Statement

All datasets generated for this study are included in the article/Supplementary Material, further inquiries can be directed to the corresponding author.

## Author Contributions

ZX, LP, YW, and B-CT designed the experiments and analyzed the data. ZX, YW, and LP performed most of the experiments. Other co-authors assisted the experiments and discussed the results. ZX and B-CT wrote the manuscript. All authors contributed to the study conception and design, read, and approved the final manuscript.

## Conflict of Interest

The authors declare that the research was conducted in the absence of any commercial or financial relationships that could be construed as a potential conflict of interest.

## Supplementary Material

Supplementary Figure 1 Clone and linkage analysis of *Emp24* and *Emp25*.

Supplementary Figure 2 Allelism test of *emp24* and *emp25*.

Supplementary Figure 3 Transcript level analysis of *Emp25* in mutants and different organs and developing seeds in maize.

Supplementary Figure 4 The editing profiles of *nad5* in WT and *emp25-1*.

Supplementary Figure 5 cRT-PCR analysis of the 5′/3′ ends of *nad5* transcripts in WT and *emp25-1*.

Supplementary Figure 6 The assembly of the mitochondrial CIII, IV, and V and respiratory rate comparison in WT and *emp25-1*.

Supplementary Figure 7 Binding sites prediction of EMP25.

Supplementary Figure 8 Major results of *emp24*.

Supplementary Figure 9 The editing profiles of *nad4* in WT and *emp24-1*.

Supplementary Table 1 Peptides of CI identified details in MS analysis.

Supplementary Table 2 Primers used in this study.
