# Supplementary material for: Empty Pericarp24 and Empty Pericarp25 Are Required for the Splicing of Mitochondrial Introns, Complex I Assembly, and Seed Development in Maize
Source: Front Plant Sci. 2020 Dec 23;11:608550. doi: 10.3389/fpls.2020.608550 (PMC7793708; doi:10.3389/fpls.2020.608550)
Supplement: Supplementary Figure 1 — Clone and linkage analysis of Emp24 and Emp25. [file Data_Sheet_1.docx]

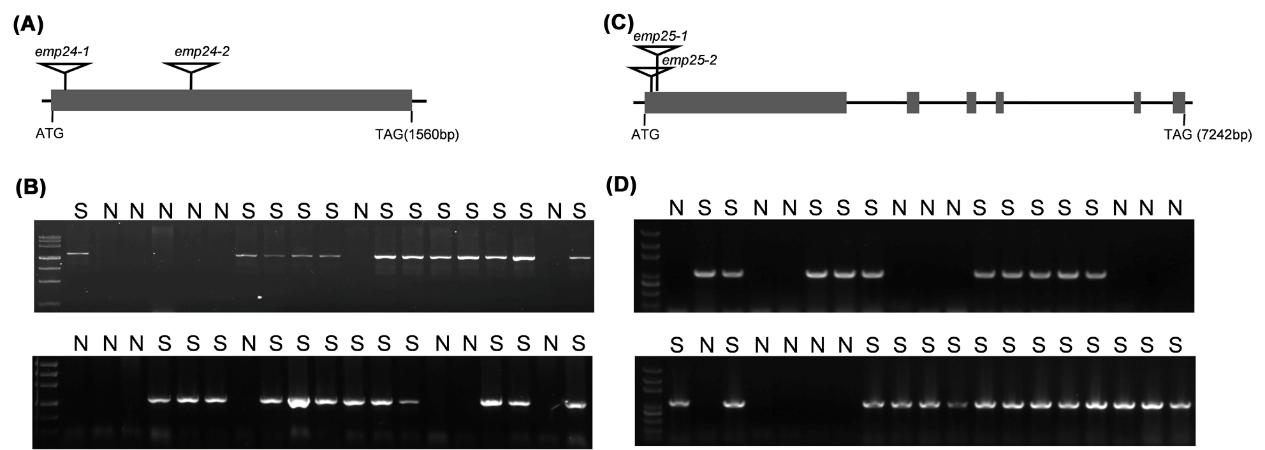


**Supplemental Figure 1. Cloning and linkage analysis of *Emp24* and *Emp25***

(A) Structure of the *Emp24* gene with *Mutator* (*Mu*) insertion (triangles) in two alleles. (C) Structure of the *Emp25* gene with insertion positions of *Mu* (triangles) in two alleles. B and D are genotype of *emp24-1* and *emp25-1* progenies, respectively. TIR8 and gene specific primers were used in PCR. N: non-segregation ear (WT); S: segregation ear (heterozygote).


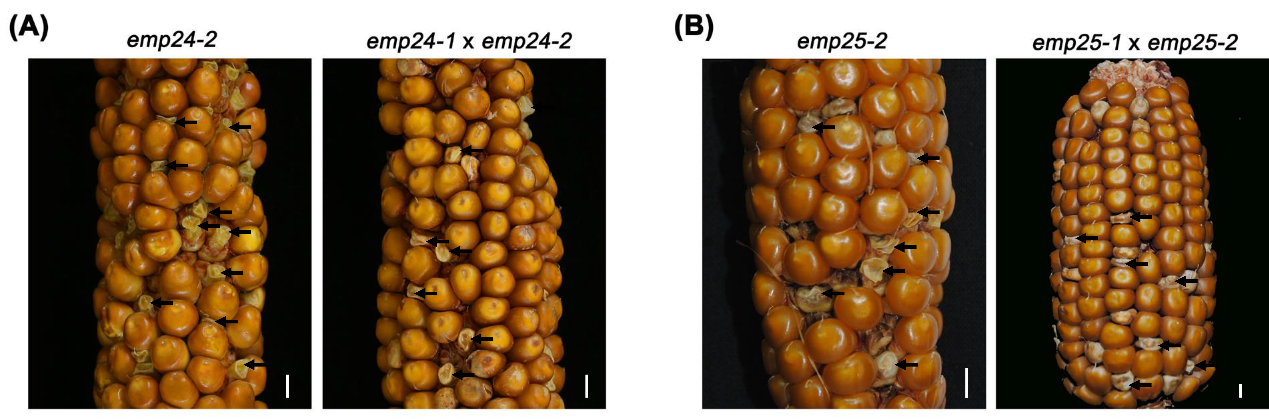


**Supplemental Figure 2. Allelism test of *emp24* and *emp25***

A, Phenotype of *emp24-2* ear and the progeny ear of two alleles cross. B, Phenotype of *emp25-2* and cross between two alleles.


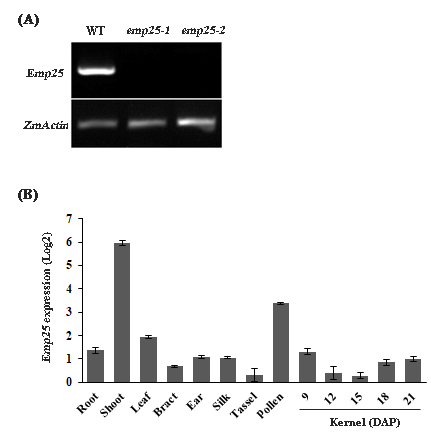


**Supplemental Figure 3. Transcript level analysis of *Emp25* in mutants and different organs and developing seeds in maize**

(A) *Emp25* expression in the wild type (WT) and two *emp25* mutant alleles. (B) Emp25 expression in different organs and developing seeds. The expression of Emp25 in 21DAP kernel is regard as 1. Values represent the mean and standard deviation of three biological replicates.


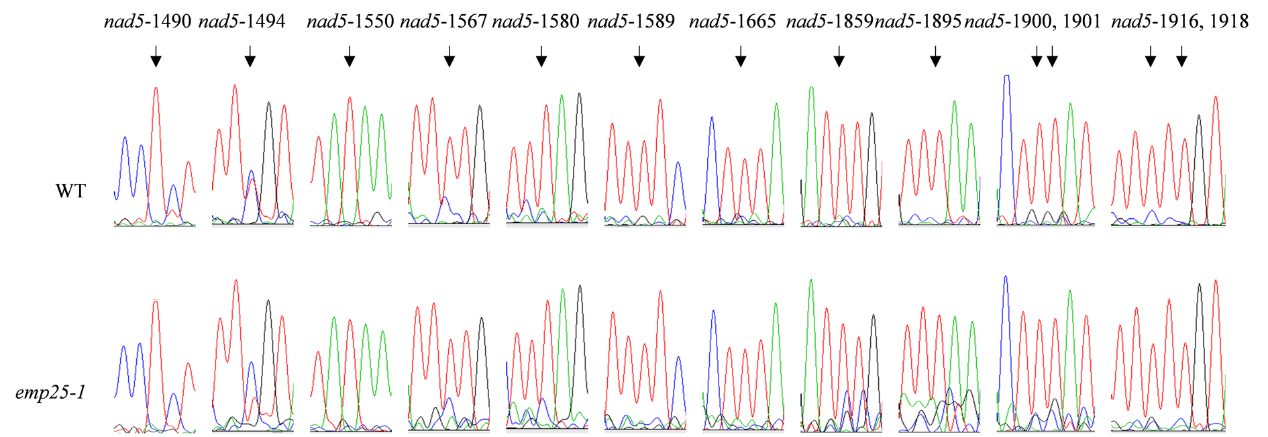


**Supplemental Figure 4. The editing profiles of *nad5* in WT and *emp25*.**

The editing sites are arrowed.


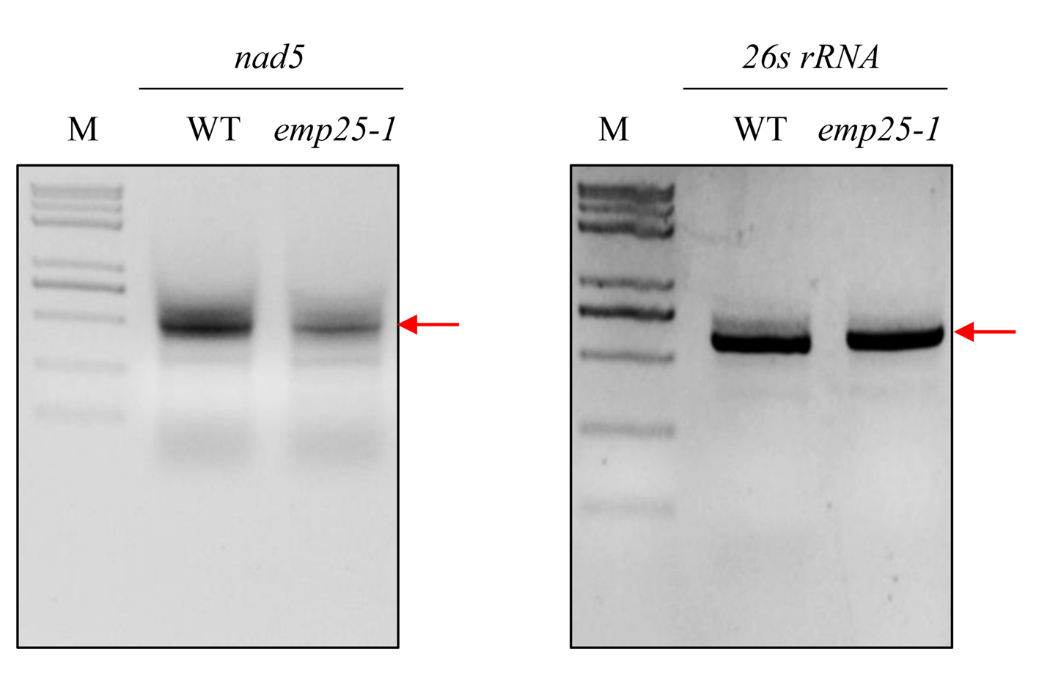


**Supplemental Figure 5. cRT-PCR analysis of the 5’ and 3’ ends of *nad5* transcripts in WT and *emp25*.**

The two samples were normalized by amplification of 26S rRNA (26S) using outward-facing primers.


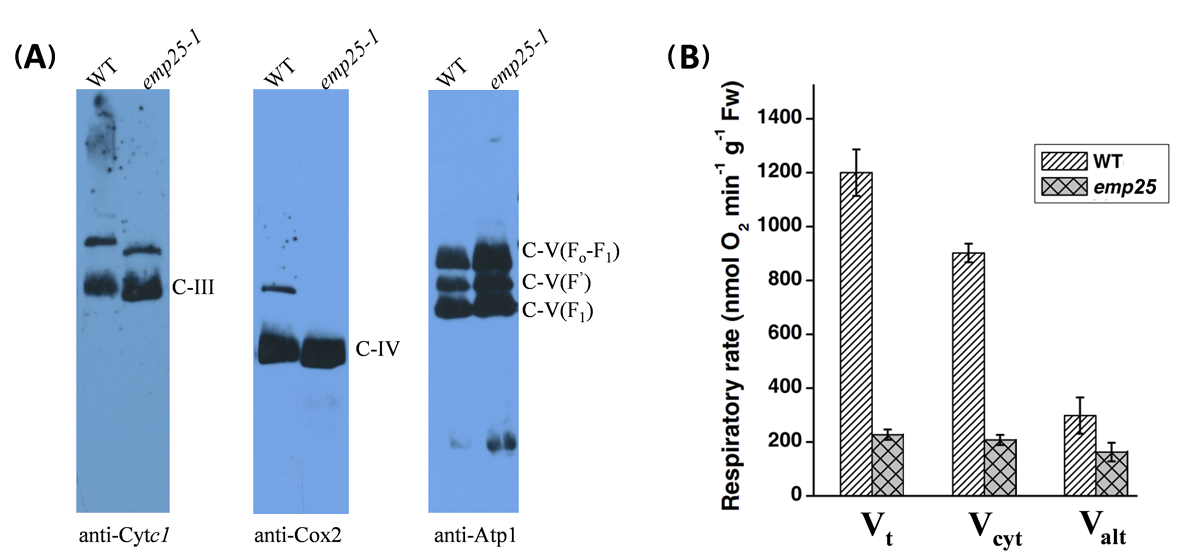


**Supplemental Figure 6. The assembly of the mitochondrial complexes III, IV and V and resporatary rate comparision in WT and *emp25*.**

1. Western blot analysis based on antibodies recognizing Cyt*c1* (Complex III, C-III), Cox2 (Complex IV, C- IV) and Atp1 (Complex V, C-V). (B) Mitochondrial total respiration rate (V_t_), the capacity of the cytochrome pathway (V_cyt_) and the alternative pathway (V_alt_) were measured using a Chlorolab II liquid-phase oxygen electrode; 2mM KCN and 2mM SHAM were used to inhibit the activity of cytochorome *c* oxidase and an alternative oxidase, respectively. Values represent the mean and standard deviation of three biological replicates. V_alt_/V_t_ indicates the contribution of alternative pathway.


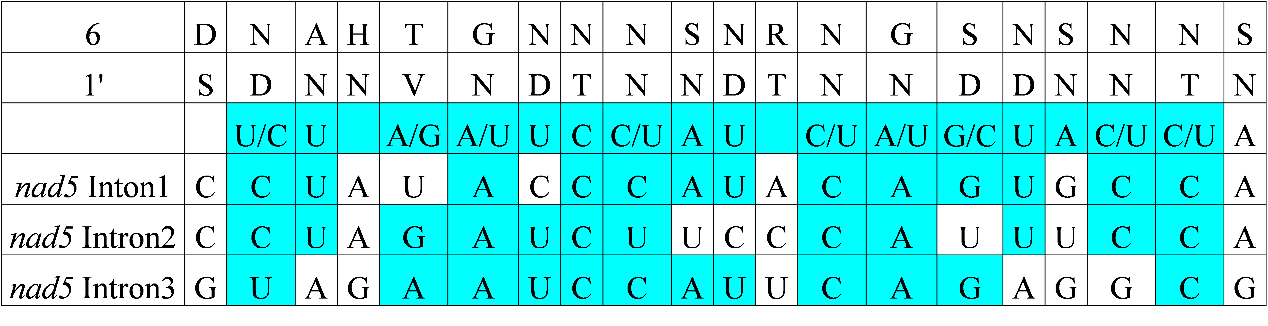


**Supplemental Figure 7. Binding site prediction of EMP25**

Alignment of the amino acid residues at position 6 and 1' in each PPR motif of EMP25 with the putative recognition sequences in *nad5* introns as descripted in (Barkan *et al*. 2012; Takenaka *et al*. 2013; Yagi *et al*. 2013; Yan *et al*. 2019).


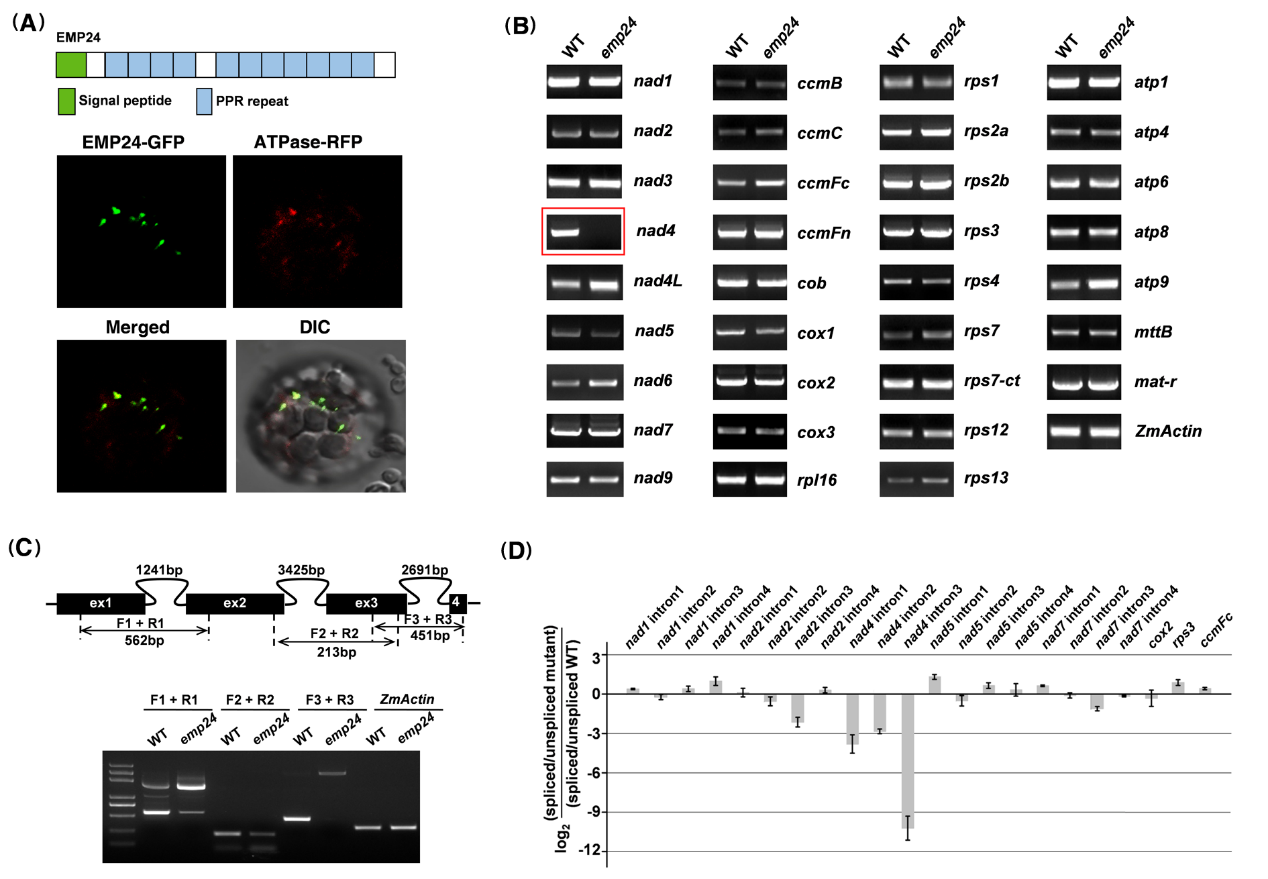


**Supplemental Figure 8. EMP24 is a PPR protein and functions in mitochondrial *nad4* intron splicing**

(A) Schematic diagram of the EMP24 proteins and subcellular localization of EMP24. Full length of *Emp24* (without stop codon) was ligated into pBI221 which fuse GFP at the N terminal. The EMP24-GFP was co-transiently expressed with ATPase-RFP ( mitochondria marker) in *Arabidopsis thaliana* leaves protoplast. The signal was detected by confocal fluorescent microscopy. Scale bar=5um. (B) Semi-quantatitive RT-PCR analysis of full length transcript levels of 34 mitochondrial protein coding genes in *emp24* with sibling WT. RNA was extracted from 13 DAP kernels after pericarp removed and was normalized against *ZmActin*. (C) ) Gene model of maize mitochondrial *nad4* gene. Three introns are *cis*-splicing intorns. The expected products size of different primer pairs amplification are indicated. Semi-quantatitive RT-PCR analysis of intron splicing efficiency in the WT and *emp24*. The arrows show the un-spliced fragments of intron1 and 3. (D) Quantitative RT-PCR (qRT-PCR) analysis of 22 group II intorns in maize mitochondrial genes in *emp24*. Values represent the mean and standard deviation of three biological replicates.


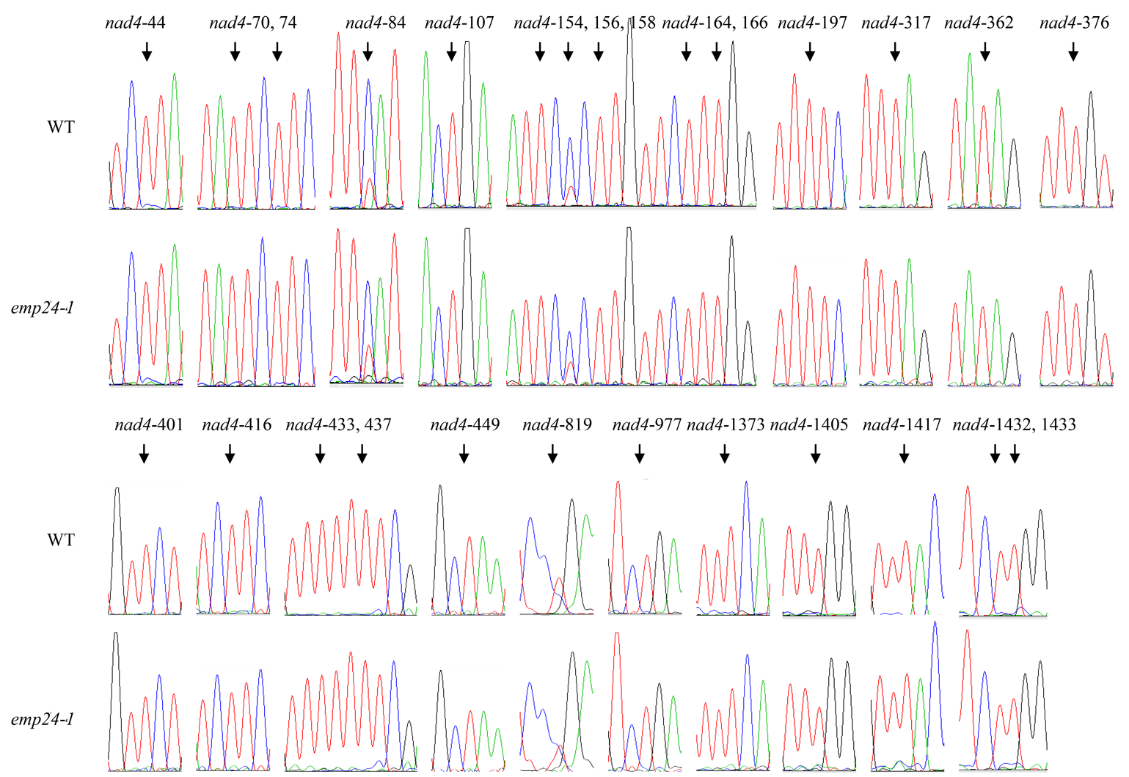


**Supplemental Figure 9. The editing profiles of *nad4* in WT and *emp24*.**

The editing sites are arrowed.
