# Supplementary material for: Empty Pericarp24 and Empty Pericarp25 Are Required for the Splicing of Mitochondrial Introns, Complex I Assembly, and Seed Development in Maize
Source: Front Plant Sci. 2020 Dec 23;11:608550. doi: 10.3389/fpls.2020.608550 (PMC7793708; doi:10.3389/fpls.2020.608550)
Supplement: Supplementary Table 1 — Peptides of CI identified details in MS analysis. [file Table_1.DOC]

**Table S1. Primers used in this study.**

| **Primer name** | **Primer sequence (5’ to 3’)** | **Use for** |
| --- | --- | --- |
| TIR8.1 | CGCCTCCATTTCGTCGAATCCCCTS | Genotype *emp24* and *emp25* mutants |
| TIR8.2 | CGCCTCCATTTCGTCGAATCCSCTT | Genotype *emp24* and *emp25* mutants |
| TIR8.3 | SGCCTCCATTTCGTCGAATCCCKT | Genotype *emp24* and *emp25* mutants |
| TIR8.4 | CGCCTCCATTTCGTCGAATCACCTC | Genotype *emp24* and *emp25* mutants |
| Emp24-F1 | CGACACCAAATACCCGGACC | Genotype *emp24* mutant |
| Emp25-R1 | GATTGAGCCAAGCGAGCA | Genotype *emp25* mutant |
| EMP25-CF1 | CACCATGGCGCGAAGCAACAGCTG | Construct full length EMP25^223aa^: GFP vector |
| EMP25-CR1 | GTCCCCAGTCTGTGCTAACCT | Construct full length EMP25^223aa^: GFP vector |
| nad5-F1 | CGCTCGAACATTGTCTGATT | Test *nad5* intron 1 splicing efficiency by RT-PCR |
| nad5-R1 | GCCGGGATCATTAAGAGCATAC |
| nad5-F2 | GTCACTGCTGGCGTTTTC | Test *nad5* intron 2 splicing efficiency by RT-PCR |
| nad5-R2 | TACCTAAACCAATCATCATATC |
| nad5-F3 | GATATGATGATTGGTTTAGGTA | Test *nad5* intron 3 splicing efficiency by RT-PCR |
| nad5-R3 | GCCAATCGTCGGAATGTG |
| nad5-F4 | TTGCCGAATCCGAGTTTG | Test *nad5* intron 4 splicing efficiency by RT-PCR |
| nad5-R4 | GTCCTGGCAAGCTCCTACAG |
| nad1exon1F | GCAACGTCGAAAGGGTCCTG, | Test *nad1* intron 1 splicing efficiency |
| nad1-intron1-F | GGTATAGAGCCGTAAGCGCG |
| nad1exon2R | TGAGCTGCAGATCGTAATGC |
| nad1exon2F | TCGAAATATGCCTTTCTAGGAG | Test *nad1* intron 2 splicing efficiency |
| nad1-intron2-F | AGCACGGACGAGCCACAT |
| nad1exon3R | ATTCAGCTTCCGCTTCTGG |
| nad1exon3F | GTCATGGCGCAAAAGCAGATATGG | Test *nad1* intron 3 splicing efficiency |
| nad1-intron3-F | ACTACTATACTACTGCGAGCGGGA |
| nad1exon4R | AGAGCAGACCCCATTGAAGA |
| nad1exon4F | TCTTCAATGGGGTCTGCTCT | Test *nad1* intron 4 splicing efficiency |
| nad1-intron4-F | CACGGAGCTGCATCCCTACT |
| nad1exon5R | AGGGAGCCATCGAAAGGTGA |
| nad2exon1F | GACGGAGGAGAGGAAATGAA | Test *nad2* intron 1 splicing efficiency |
| nad2-intron1-F | TATCACGGACGAGCCACATG |
| nad2exon2R | GCCGGGATCATTAAGAGCATAC |
| nad2exon2F | CTCGCAGTATGCTCTTAATGATCC | Test *nad2* intron 2 splicing efficiency |
| nad2-intron2-F | GAGCCGTATGCGGTGAGA |
| nad2exon3R | GGAACTGCAGTAATCTTGAATAGGG |
| nad2-exon3F | TCTACTGGAGCTACCCACTTCGA | Test *nad2* intron 3 splicing efficiency |
| nad2-intron3-F | AAGGCAGCCATCCTCATAAAG |
| nad2-exon4R | GGTTTGCCGTAATGCTGGA |
| nad2exon4F | TTCCAGCATTACGGCAAACC | Test *nad2* intron 4 splicing efficiency |
| nad2-intron4-F | CGTATGATGGGCAACTATCTCCT |
| nad2exon5R | GCAGTCCACCCTTTCTTTGA |
| nad4exon1F | GGTCCTATTCTCTGTCCCGTGC | Test *nad4* intron 1 splicing efficiency |
| nad4-intron1-F | GAGCCGTATGATGCGGAAGT |
| nad4exon2R | GTAAATCGGTGGTTCCTGTTTGG |
| nad4exon2F | TCATTATAGGGGTATGGGGTTCG | Test *nad4*intron 2 splicing efficiency |
| nad4-intron2-F | GAAAGCCGTATGATAGGTGGTAAC |
| nad4exon3R | CTAGTGCCGGGTAAACTCATATTG |
| nad4exon3F | TAGTCCGAACATACCGGGAATTG | Test *nad4* intron 3 splicing efficiency |
| nad4-intron3-F | GTCCAGGTTGGTTGGTGAGC |
| nad4exon4R | CTTACGGATGTATGCATGCAGTC |
| nad5exon1F | CGCTCGAACATTGTCTGATT | Test *nad5* intron 1 splicing efficiency |
| nad5-intron1-F | GTTCACCACCACATCATTGCA |
| nad5exon2R | AGCAGATACTGGAGTGGGAC |
| nad5exon2F | GTCACTGCTGGCGTTTTC | Test *nad5* intron 2 splicing efficiency |
| nad5-intron2-F | GGAGGGCTTTGTTTGTGCA |
| nad5exon3R | TACCTAAACCAATCATCATATC |
| nad5exon3F | GATATGATGATTGGTTTAGGTA | Test *nad5* intron 3 splicing efficiency |
| nad5-intron3-F | GCAGAGTTTGTGAGCCGTGTAA |
| nad5exon4R | GCCAATCGTCGGAATGTG |
| nad5exon4F | TTGCCGAATCCGAGTTTG | Test *nad5* intron 4 splicing efficiency |
| nad5-intron4-F | GTGGTAAAGGGAGGGAGGATATT |
| nad5exon5R | GTCCTGGCAAGCTCCTACAG |
| nad7exon1F | TAATTTGGCGCCTGATTGAC | Test *nad7* intron 1 splicing efficiency |
| nad7-intron1-F | GGATTTGCGAATGAATGCTG |
| nad7exon2R | CTCGATTAATTTCTCAGTCCCTC |
| nad7exon2F | GAGGGACTGAGAAATTAATCGAG | Test *nad7* intron 2 splicing efficiency |
| nad7-intron2-F | GTTGTTCGTTCCGTCGTTGA |
| nad7exon3R | CTCGACATAAGCCAAGAGGC |
| nad7exon3F | GCCTCTTGGCTTATGTCGAG | Test *nad7* intron 3 splicing efficiency |
| nad7-intron3-F | AGCCGTATGAAGGGAAACTCC |
| nad7exon4R | CCGAACACTTTGTCGCATCT |
| nad7exon4F | AGATGCGACAAAGTGTTCGG | Test *nad7* intron 4 splicing efficiency |
| nad7-intron4-F | ACCGGATCATCGGTCTACTCTA |
| nad7exon5R | GTTTTGGCTCGCAATAAAGC |
| ccmFCexon1F | CGATAGGTCAGCGAAGCGTG | Test *ccmFc* intron splicing efficiency |
| ccmFC-intron-F | GAGGCAGAAACTCGTCCCAC |
| ccmFCexon1R | AGACCTCGCAAACAACAACGT |
| cox2-exonF1 | GCTCTGTTATACTCAATGGACGGG | Test *cox2* intron splicing efficiency |
| cox2-intron-F | AAATAAGAGTAGGCGTGGAGAGC |
| cox2-exonR1 | AGATGAGTTTTGGCTGGTACAACC |
| rps3-exonF1 | TTTCGGTAAGACTTGATCTGAATCG | Test *rps3* intron splicing efficiency |
| rps3-intron-F | ACCACAGGTTGGGTTTGAGAG |
| rps3-exonR1 | TATCCTTTCCGGGTCTTGATTTGTC |
| nad5-ex1-F | CGCTCGAACATTGTCTGATT | Test *nad5* intron2 and 3 mis-splicing |
| nad5-in1-F | GTTCACCACCACATCATTGCA |  |
| nad5-ex2-R | AGCAGATACTGGAGTGGGAC |  |
| nad5-ex2-F | CGGGCGAGACAGATTACGAT |  |
| nad5-in2-R | TCATATCTTTGGCCAAGTATCCTAC |  |
| nad5-in2-F | GGAGGGCTTTGTTTGTGCA |  |
| nad5-ex3-F | GATATGATGATTGGTTTAGGTA |  |
| nad5-ex3-R | TACCTAAACCAATCATCATATC |  |
| nad5-in3-R | CGGTCTCACGCACTAATCCC |  |
| nad5-in3-F | GCAGAGTTTGTGAGCCGTGTAA |  |
| nad5-ex4-R | GCCAATCGTCGGAATGTG |  |
| nad5-ex4-F | TTGCCGAATCCGAGTTTG |  |
| nad5-in4-F | GTGGTAAAGGGAGGGAGGATATT |  |
| nad5-ex5-R | GTCCTGGCAAGCTCCTACAG |  |
